# Emergency Department Utilization for Hypertensive Disorders of Pregnancy and Post Partum, 2006-2020

**DOI:** 10.1001/jamanetworkopen.2024.33045

**Published:** 2024-09-13

**Authors:** Courtney Townsel, LeAnn Louis, Chelsie Clark, Leah Mitchell Solomon, Charley Jiang, Martina Caldwell, Erica E. Marsh

**Affiliations:** 1 Division of Maternal Fetal Medicine, Department of Obstetrics, Gynecology and Reproductive Sciences, University of Maryland, Baltimore; 2 Department of Obstetrics and Gynecology, University of Michigan, Ann Arbor; 3 University of Michigan Medical School, Ann Arbor; 4 Division of Women’s Health, Department of Obstetrics and Gynecology, University of Michigan, Ann Arbor; 5 Division of Reproductive Endocrinology and Infertility, Department of Obstetrics and Gynecology, University of Michigan, Ann Arbor; 6 Department of Emergency Medicine, Henry Ford Hospital, Detroit, Michigan; 7 Department of Women’s and Gender Studies, College of Literature, Sciences, and the Arts, University of Michigan, Ann Arbor; 8 Michigan Institute of Clinical and Health Research, University of Michigan, Ann Arbor

## Abstract

This cross-sectional study assesses emergency department (ED) admissions for hypertensive disorders of pregnancy and post partum between 2006 and 2020.

## Introduction

Hypertensive disorders of pregnancy and the postpartum period (HDPP) are the second leading cause of maternal deaths worldwide^1,2^ and account for 6.3% of all pregnancy-related deaths in the US.^3^ Black birthing people have the highest rates of pregnancy-related mortality in the US.^4^ The American College of Obstetricians and Gynecologists recommends management of severe blood pressure in pregnancy within 30 to 60 minutes of diagnosis to prevent complications such as stroke, myocardial ischemia, seizure, placental abruption, and maternal and neonatal mortality.^5^ Given the need for prompt intervention, the emergency department (ED) is a critical access point for treatment of HDPP. To our knowledge, there are no studies reporting national trends of ED use for HDPP. In this study, we assess US ED utilization and admission for HDPP.

## Methods

This cross-sectional study was determined exempt from review and the requirement of informed consent by the University of Michigan institutional review board and followed the STROBE reporting guidelines. This was a longitudinal retrospective analysis of HDPP-related ED utilization from 2006 to 2020 using the Nationwide Emergency Department Sample (NEDS), managed by the Healthcare Cost and Utilization Project of the Agency for Healthcare Research and Quality. Pregnant or postpartum people aged 15 to 50 years with a primary diagnosis of HDPP by *International Classification of Diseases, Ninth Revision (ICD-9)* or *International Statistical Classification of Diseases and Related Health Problems, Tenth Revision (ICD-10)* codes were included (eMethods in Supplement 1). The primary outcome was total annual ED visits for HDPP. Secondary outcomes included hospital admissions, and HDPP-related ED use by race and ethnicity. Characteristics including age, income quartile by zip code, payment method, hospital geographic region, and hospital teaching status were analyzed. Race and ethnicity data, abstracted from the NEDS database, were only available for 2019 to 2020. Race and ethnicity categories included Asian or Pacific Islander, non-Hispanic Black, Hispanic, American Indian or Alaska Native, non-Hispanic White, and other (defined as multiracial).

Considering the complex sample design, SAS survey sampling and analysis procedures were applied, including stratum, clusters, and weights into χ^2^, *t* tests, and multivariable logistic regression. Statistical significance was defined as a 2-sided *P* < .05. SAS version 9.4 (SAS Institute) was used for analysis. Analysis occurred from August 2022 to February 2023.

## Results

Between 2006 and 2020, 656 711 HDPP-related ED encounters occurred. HDPP annual ED visits increased from 31 623 to 55 893 from 2006 to 2020 (*P* < .001) (Table 1). Admissions and admission rates for HDPP also increased over the study period from 17 338 admissions in 2006 (rate, 54.8%) to 43 563 admissions in 2020 (rate, 77.9%) (*P* < .001), in contrast with stable admission rates for all other primary diagnoses (2 823 749 or 35 177 059 admissions [8.0%] in 2006 to 2 796 555 of 33 402 798 admissions [8.4%] in 2020; *P* = .08).

**Table 1.** Characteristics of Emergency Department Visits for Primary Diagnosis of Hypertension

Characteristic	Emergency department visits for HDPP by year, No. (%) (N = 371 292)							
	2006 (n = 31 623)	2008 (40 247)	2010 (n = 46 974)	2012 (n = 45 275)	2014 (n = 61 677)	2016 (n = 47 520)	2018 (n = 42 083)	2020 (n = 55 893)
Age group, y								
15-19	3938 (12.5)	4739 (11.8)	4261 (9.1)	3114 (6.9)	3898 (6.3)	2989 (6.3)	2384 (5.7)	2399 (4.3)
20-24	7839 (24.8)	9271 (23.0)	10 617 (22.6)	9418 (20.8)	11 877 (19.3)	8969 (18.9)	7952 (18.9)	8811 (15.8)
25-29	8137 (25.7)	9951 (24.7)	11 807 (25.1)	11 848 (26.2)	16 005 (25.9)	12 373 (26.0)	10 459 (24.9)	13 406 (24.0)
30-34	6392 (20.2)	8693 (21.6)	10 964 (23.3)	11 409 (25.2)	16 000 (25.9)	12 619 (26.6)	11 202 (26.6)	16 096 (28.8)
35-39	3947 (12.5)	5645 (14.0)	6389 (13.6)	6996 (15.5)	10 415 (16.9)	7991 (16.8)	7549 (17.9)	11 429 (20.4)
40-44	1287 (4.1)	1776 (4.4)	2653 (5.6)	2264 (5.0)	3208 (5.2)	2391 (5.0)	2351 (5.6)	3382 (6.1)
45-50	82 (0.3)	172 (0.4)	283 (0.6)	227 (0.5)	272 (0.4)	189 (0.4)	186 (0.4)	371 (0.7)
Region								
Northeast	3799 (12.0)	12 727 (31.6)	10 487 (22.3)	12 738 (28.1)	15 469 (25.1)	9297 (19.6)	9308 (22.1)	14 060 (25.2)
Midwest	5790 (18.3)	6885 (17.1)	7690 (16.4)	5810 (12.8)	8492 (13.8)	7741 (16.3)	7881 (18.7)	7699 (13.8)
South	17 542 (55.5)	17 127 (42.6)	22 398 (47.7)	21 774 (48.1)	29 480 (47.8)	24 014 (50.5)	19 439 (46.2)	25 280 (45.2)
West	4492 (14.2)	3508 (8.7)	6400 (13.6)	4954 (10.9)	8236 (13.4)	6468 (13.6)	5455 (13.0)	8854 (15.8)
Urbanicity								
Metro, (≥1 million people)	19 045 (60.2)	26 247 (65.2)	32 672 (69.6)	28 568 (63.1)	40 628 (65.9)	32 389 (68.2)	27 880 (66.3)	37 752 (67.5)
Metro (50 000 to <1 million people)	7386 (23.4)	8958 (22.3)	9330 (19.9)	12 377 (27.3)	15 590 (25.3)	9636 (20.3)	10 469 (24.9)	13 092 (23.4)
Nonmetro (<50 000 people)	5131 (16.2)	4889 (12.1)	4815 (10.3)	4258 (9.4)	5304 (8.6)	5402 (11.4)	3681 (8.7)	4868 (8.7)
Primary payer								
Medicare	329 (1)	598 (1.5)	625 (1.3)	929 (2.1)	977 (1.6)	705 (1.5)	464 (1.1)	642 (1.1)
Medicaid	16 203 (51.2)	19 983 (49.7)	24 964 (53.1)	24 500 (54.1)	34 430 (55.8)	23 813 (50.1)	20 977 (49.8)	24 617 (44.0)
Private	12 753 (40.3)	15 473 (38.4)	17 186 (36.6)	16 758 (37.0)	22 152 (35.9)	20 338 (42.8)	18 825 (44.7)	27 885 (49.9)
Self-pay	1522 (4.8)	3027 (7.5)	2987 (6.4)	1692 (3.7)	1979 (3.2)	1553 (3.3)	1048 (2.5)	1292 (2.3)
No charge	64 (0.2)	84 (0.2)	36 (0.1)	95 (0.2)	106 (0.2)	98 (0.2)	23 (0.1)	53 (0.1)
Other	631 (2.0)	902 (2.2)	1067 (2.3)	1262 (2.8)	1167 (1.9)	997 (2.1)	747 (1.8)	1256 (2.2)
Income quartile by zip code								
1	11 390 (36.0)	14 909 (37.0)	17 585 (37.4)	15 526 (34.3)	22 518 (36.5)	16 664 (35.1)	14 589 (34.7)	16 378 (29.3)
2	8746 (27.7)	9869 (24.5)	11 360 (24.2)	11 473 (25.3)	16 084 (26.1)	11 419 (24.0)	10 508 (25.0)	13 620 (24.4)
3	6681 (21.1)	7799 (19.4)	10 023 (21.3)	10 804 (23.9)	11 544 (18.7)	10 784 (22.7)	9276 (22.0)	12 792 (22.9)
4	4371 (13.8)	5047 (12.5)	5881 (12.5)	6646 (14.7)	8761 (14.2)	8168 (17.2)	7480 (17.8)	12 673 (22.7)
Admission rate								
HDPP	17 338 (54.8)	22 467 (55.8)	26 963 (57.4)	22 220 (49.1)	33 123 (53.7)	19 776 (80.2)	33 729 (80.1)	43 563 (77.9)
Other primary diagnoses, No./total No. (%)	2 823 749/35 177 059 (8.0)	2 994 604/37 067 603 (8.1)	3 046 591/38 838 100 (7.8)	2 839 693/39 404 810 (7.2)	2 872 802/40 291 049 (7.1)	2 711 703/41 067 859 (6.6)	2 790 118/38 951 593 (7.2)	2 796 555/33 402 798 (8.4)

Abbreviation: HDPP, hypertensive disorder of pregnancy and the postpartum period.

Non-Hispanic Black, Hispanic, and Asian or Pacific Islander individuals were more likely to present to the ED for HDPP compared with all other diagnoses (non-Hispanic Black, 27 968 of 104 556 visits [26.7%] vs 17 942 147 of 70 664 334 visits [25.4%]; *P* = .007; Hispanic, 22 097 of 104 556 visits [21.1%] vs 12 405 817 of 70 664 334 visits [17.6%]; *P* < .001; and Asian or Pacific Islander, 4603 of 104 556 visits [4.4%] vs 1514913 of 70 664 334 visits [2.1%]; *P* < .001). Additionally, compared with non-Hispanic White individuals, non-Hispanic Black, Hispanic, and Asian or Pacific Islander individuals were more likely to be admitted for HDPP (Table 2).

**Table 2.** Hospital Admission With Hypertensive Disorders in Pregnancy and Postpartum by Race and Ethnicity, 2019-2020

Race and ethnicity	Hospital admission with hypertensive disorder of pregnancy, adjusted OR (95% CI)^a^	*P* value
American Indian or Alaska Native	0.66 (0.37-1.18)	.16
Asian or Pacific Islander	1.40 (1.09-1.80)	.009
Hispanic	1.66 (1.41-1.95)	<.001
Non-Hispanic Black	1.16 (1.02-1.32)	.02
Non-Hispanic White	1 [Reference]	NA
Other^b^	1.13 (0.89-1.44)	.32

Abbreviations: NA, not applicable; OR, odds ratio.

^a^ Multivariable regression model adjusted for income quartile, insurance type, hospital teaching status, and metropolitan size.

^b^ Other was defined as 2 or more races or multiracial.

## Discussion

In this cross-sectional study, US ED visits and admissions for HDPP increased significantly from 2006 to 2020. This finding may reflect a higher prevalence of disease or increased awareness for prompt assessment and treatment. However, the greater ED utilization for HDPP compared with all other diagnoses for non-Hispanic Black, Hispanic, and Asian or Pacific Islander individuals may imply limited access to timely outpatient care or barriers to uptake blood pressure monitoring programs. Furthermore, non-Hispanic Black, Hispanic, and Asian or Pacific Islander individuals were more likely to be admitted for HDPP than non-Hispanic White individuals, suggesting worse disease severity at presentation.

Study strengths include a nationally representative dataset with large sample and inclusion of recently added race and ethnicity data. Limitations include a visit-based dataset that may count individuals multiple times and use of diagnostic codes which shifted from *ICD-9* to *ICD-10* between 2015 and 2016. Racial differences in ED utilization for HDPP underscore the ongoing racial disparities in US maternal morbidity and mortality and highlight a critical need for accessible, culturally competent community-level interventions for all.

## Notes

Supplement 1. **eMethods.** Diagnostic Codes

Supplement 2.
Data Sharing Statement
