# Supplementary material for: Emergency Department Utilization for Hypertensive Disorders of Pregnancy and Post Partum, 2006-2020
Source: JAMA Netw Open. 2024 Sep 13;7(9):e2433045. doi: 10.1001/jamanetworkopen.2024.33045 (PMC11400215; doi:10.1001/jamanetworkopen.2024.33045)
Supplement: Supplement 1. — eMethods. Diagnostic Codes [file jamanetwopen-e2433045-s001.pdf]

Supplemental Online Content

Townsel C, Louis L, Clark C, et al. Emergency department utilization for hypertensive disorders of pregnancy and postpartum, 2006-2020. *JAMA Netw. Open*. 2024;7(9):e2433045. doi:10.1001/jamanetworkopen.2024.33045

eMethods. Diagnostic Codes

This supplemental material has been provided by the authors to give readers additional information about their work.

© 2024 Townsel C et al. *JAMA Network Open*.

eMethods. Diagnostic Codes

ICD9		ICD10	
**Code**	**Description**	**Code**	**Description**
642.0x	Benign essential hypertension complicating pregnancy, childbirth, and the puerperium	O14.0x	Mild to moderate pre-eclampsia
642.1x	Hypertension secondary to renal disease, complicating pregnancy, childbirth, and the puerperium	O14.1x	Severe pre-eclampsia
642.2x	Other pre-existing hypertension complicating pregnancy, childbirth, and the puerperium	O14.2x	HELLP syndrome
642.3x	Transient hypertension of pregnancy	O14.9x	Unspecified pre-eclampsia
642.9x	Unspecified hypertension complicating pregnancy, childbirth, and the puerperium	O11.x	Pre-existing hypertension with pre-eclampsia
642.4x	Mild or unspecified pre-eclampsia		
642.5x	Severe pre-eclampsia		
642.6x	Eclampsia		
642.7x	Pre-eclampsia or eclampsia superimposed on pre-existing hypertension		

© 2024 Townsel C et al. *JAMA Network Open*.
