# Supplementary material for: Emergency Department Utilization for Hypertensive Disorders of Pregnancy and Post Partum, 2006-2020
Source: JAMA Netw Open. 2024 Sep 13;7(9):e2433045. doi: 10.1001/jamanetworkopen.2024.33045 (PMC11400215; doi:10.1001/jamanetworkopen.2024.33045)
Supplement: Supplement 2. — Data Sharing Statement [file jamanetwopen-e2433045-s002.pdf]

Data Sharing Statement

Townsel. Emergency Department Utilization for Hypertensive Disorders of Pregnancy and Postpartum, 2006-2020. *JAMA Netw Open*. Published September 13, 2024. doi:10.1001/jamanetworkopen.2024.33045

Data
Data available: No

Additional Information
Explanation for why data not available: The data are publicly available for purchase from HCUP, but per their contract, we cannot share their data.
